# Blue-light filtering alters angiogenic signaling in human retinal pigmented epithelial cells culture model

**DOI:** 10.1186/s12886-017-0592-2

**Published:** 2017-11-02

**Authors:** Natalia Vila, Aya Siblini, Evangelina Esposito, Vasco Bravo-Filho, Pablo Zoroquiain, Sultan Aldrees, Patrick Logan, Lluis Arias, Miguel N. Burnier

**Affiliations:** 1 Henry C. Witelson Ocular Pathology Laboratory, Pathology Department, McGill University, Montreal, Canada; 2 Hospital Universitari de Bellvitge, Ophthalmology Department, Barcelona University, Barcelona, Spain

**Keywords:** Blue light filters, RPE cell line, Angiogenesis, Age-related macular degeneration

## Abstract

**Background:**

Light exposure and more specifically the spectrum of blue light contribute to the oxidative stress in Age-related macular degeneration (AMD). The purpose of the study was to establish whether blue light filtering could modify proangiogenic signaling produced by retinal pigmented epithelial (RPE) cells under different conditions simulating risk factors for AMD.

**Methods:**

Three experiments were carried out in order to expose ARPE-19 cells to white light for 48 h with and without blue light-blocking filters (BLF) in different conditions. In each experiment one group was exposed to light with no BLF protection, a second group was exposed to light with BLF protection, and a control group was not exposed to light. The ARPE-19 cells used in each experiment prior to light exposure were cultured for 24 h as follows: Experiment 1) Normoxia, Experiment 2) Hypoxia, and Experiment 3) Lutein supplemented media in normoxia. The media of all groups was harvested after light exposure for sandwich ELISA-based assays to quantify 10 pro-angiogenic cytokines.

**Results:**

A significant decrease in angiogenin secretion levels and a significant increase in bFGF were observed following light exposure, compared to dark conditions, in both normoxia and hypoxia conditions. With the addition of a blue light-blocking filter in normoxia, a significant increase in angiogenin levels was observed. Although statistical significance was not achieved, blue light filters reduce light-induced secretion of bFGF and VEGF to near normal levels. This trend is also observed when ARPE-19 cells are grown under hypoxic conditions and when pre-treated with lutein prior to exposure to experimental conditions.

**Conclusions:**

Following light exposure, there is a decrease in angiogenin secretion by ARPE-19 cells, which was abrogated with a blue light - blocking filter. Our findings support the position that blue light filtering affects the secretion of angiogenic factors by retinal pigmented epithelial cells under normoxic, hypoxic, and lutein-pretreated conditions in a similar manner.

## Background

Age-related macular degeneration (AMD) is the leading cause of severe and irreversible loss of vision in the elderly in developed countries [1]. AMD is a complex disease associated with many environmental, lifestyle, and genetic factors [2–4]. Although some aspects of AMD pathogenesis are not well understood, there is a growing body of evidence suggesting that oxidative stress can contribute to the onset and progression of the condition [5]. A known cause of such oxidative stress is exposure to short wavelength light, including ultraviolet and blue light.

Blue light exposure has been linked to abnormal secretion of potent pro-angiogenic factors that can promote the onset or progression of ocular diseases with a neovascular component. The blue light spectrum of natural sunlight is often cited as the primary cause of blue light-induced damage within the eye. Indeed, data from epidemiologic analyses support this hypothesis: the Beaver Dam Eye Study and the Chesapeake Bay Waterman Study both reported a significant association between cumulative sunlight exposure and AMD [6–9]. Moreover, it has been demonstrated that blue light-induced damage in retinal pigmented epithelial (RPE) cells can promote angiogenic signaling, thereby contributing to an environment that promotes neovascular disease [10].

The mounting evidence that blue light exposure may contribute to the development of AMD has led to the introduction of blue light-filtering intraocular lenses (IOLs) as an alternative to traditional IOL models. Previous studies have shown that blue light-filtering IOLs can mitigate light-induced increases in vascular endothelial growth factor (VEGF) compared with IOLs that only filter ultraviolet (UV) light [11, 12].

The local environment in the macula of patients with non-exudative AMD is known to be hypoxic [13]. Moreover, aging in general has been associated with inflammation and hypoxia in the choriocapillaris, RPE layer, and neurosensory retina [14]. Hypoxia may also be caused by confluent drusen— characteristic feature of AMD —, which can increase the distance between Bruch’s membrane and the RPE and neural retina, thereby impeding oxygen delivery to the latter [4, 15, 16].

Lutein is a well described, naturally occurring macular pigment; high levels of lutein in the macula are known to be protective against AMD [17, 18]. A number of studies—including the Age-Related Eye Disease Study 2 (AREDS2), a large-scale clinical trial—have demonstrated that lutein supplementation may be protective against progression from AMD to a more severe stage of the disease [19]. Recommendations on the heels of AREDS2 have led many clinicians to advise high-risk AMD patients to consume lutein-containing supplements.

Several studies suggest that filtering blue light may reduce oxidative stress and angiogenic signaling in the retina [10–12]; however, whether or not the consequences of such filtering would be altered by a hypoxic environment—such as that seen in the eyes of some AMD patients—are unknown. Similarly, it is not known whether lutein supplementation in high-risk non-exudative AMD patients can alter the well-studied benefits of blue light filtering in AMD patients. Accordingly, the purpose of the current study was to establish the degree to which blue light filtering could affect angiogenic signaling in RPE cells under normoxic, hypoxic, and lutein-treated conditions.

## Methods

Three experiments were carried out in order to expose ARPE-19 cells to white light with and without blue light-blocking filters (BLF) in different conditions (normoxia, hyperoxia and lutein pre-treated). Each experiment had one group exposed to light with a BLF protection, a second group exposed to light with no filter protection, and a light-unexposed control group. The ARPE-19 cells used in each experiment prior to light exposure were cultured for 24 h, as described below. The media of all groups was harvested after light exposure to quantify pro-angiogenic cytokines.

### Cell culture

The ARPE-19 cell line was obtained from American Type Culture Collection (Manassas, VA, USA). Cells were incubated at 37 °C in a humidified 5% CO_2_-enriched atmosphere, and cultured in a 1:1 mixture of Dulbecco’s modified Eagle’s medium (DMEM) and Ham’s F12 medium (ThermoFisher, MA, USA) supplemented with 10% fetal bovine serum (FBS), 1% fungizone, and 1% penicillin-streptomycin. Normal growth was confirmed at every media change by phase contrast microscopy. Cultures were grown to confluence and subcultured using 0.05% trypsin in EDTA (Corning Inc., NY, USA). ARPE-19 cells at passage seven were used in all experiments.

Cells were seeded on 48-well plates at a density of 2.5 × 10^4^ cells/well for all experiments and grown in complete media overnight. The following day, cells were serum-starved and incubated for an additional 24 h in normoxia (experiment 1), hypoxia (experiment 2) or pre-treated with Lutein (experiment 3). Cells were then exposed to light with or without filter. The control plate was wrapped in aluminum foil (dark maintained), while the other two plates were left unwrapped under a light source (light exposed). Supernatants from duplicate wells were pooled and experiments were conducted in triplicate.

### Experimental conditions

*Experiment 1: Normoxic conditions -* Cells were seeded and the following day were serum-starved and incubated under normoxic conditions (37 °C, 5% CO_2_) for an additional 24 h before being exposed to light for 48 h.

*Experiment 2: Hypoxic conditions* - ARPE-19 cell cultures were serum-starved for 24 h and then incubated in 1% O_2._ Hypoxia was induced using a Modular Incubator Chamber (Billups-Rothenberg, Inc., San Diego, CA, USA) before being exposed to light. The hypoxic mixture of the certified medical pre-mixed gas used was: 1.00% O_2_, 4.98% CO_2_ and balance N_2_.

*Experiment 3: Pre-treatment with lutein* - Serum-free 1:1 DMEM:Ham’s F12 media supplemented with lutein crystals (Sigma-Aldrich, St. Louis, MO, USA) was prepared. Lutein crystals were dissolved in 0.05% dichloromethane solution at a final concentration of 1 μg/mL, as described in the literature [19]. ARPE-19 cell cultures were serum-starved for 24 h and then pre-treated with lutein-supplemented media. The following day were exposed to light.

### Light source and blue light filter setup

Controlled exposure of RPE cells to white light was achieved using a cell culture incubator that enabled the installation of a fixed light source (white light LED, NSSW157AT, Nichia Corporation, Japan). In order to set up the third treatment condition, a blue light filter (BLF) (R4590, Roscolux, USA; 98% blocking at 450 nm) was placed between the cell culture plate and the light source. The intensity of light reaching the surface of the cell culture plates was then measured using a luxometer and found to be an average of 9000 lx (range: 8800–9200 lx). In the group exposed to white light without a BLF, a series of transparent filters were used to reduce the intensity of light at the surface of cell culture plates to within the range measured for the group with the BLF.

### Angiogenesis arrays

Quantification of angiogenic cytokines within the collected supernatant was performed as per the manufacturer’s instructions using the Quantibody Human Angiogenesis Array 1 kit (RayBiotech Inc., Norcross, GA, USA), which is based on a sandwich enzyme-linked immunosorbent assay-based system. The array allows for the simultaneous quantification of the following 10 pro-angiogenic cytokines: angiogenin, ANG-2, EGF, bFGF, HB-EGF, PDGF-BB, Leptin, PIGF, HGF, and VEGF-A. Briefly, protein secretion levels of the angiogenic cytokines within the samples were analyzed by comparing the intensities of the fluorescent signals produced with the standard curve generated by standard samples in each array. Concentrations were reported in picograms per milliliter (pg/mL). A step-by-step methodology of this process has previously been described by our group [20].

### Statistical analysis

Shapiro-Wilk test was used to assess normal distributions in the levels of angiogenic factors and comparisons were performed with the Student’s t-test or Wilcoxon rank sum test, as appropriate. For comparison of two experimental groups, an unpaired, two-sample Welch’s t-test was used. To compare the three experimental groups, the nonparametric Kruskal-Wallis test was used, followed by Dunn’s post hoc test to calculate the *p-*value for each group. Graphs show the mean ± standard error of the mean (SEM); statistical significance was defined as *p* < 0.05. All analyses were performed using GraphPad Prism statistical software (GraphPad Software, Inc., San Diego, CA, USA).

## Results

The effect of white light exposure on the secretion of angiogenic cytokines by ARPE-19 cells was determined in three experiments. Only three of the ten cytokines analyzed were above the lower limit of detection for the assay, namely angiogenin, bFGF, and VEGF.

**Fig. 1:**
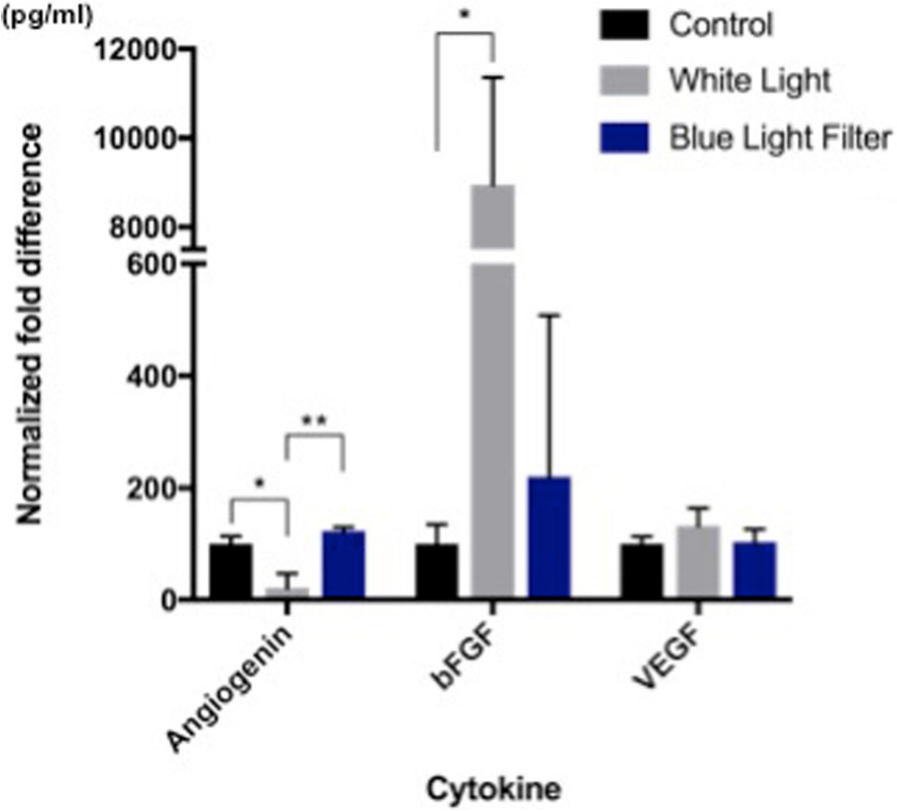
Experiment 1: Light exposure under normoxia - Secretion of angiogenin, bFGF, and VEGF by RPE cells in the dark, or under white-light exposure, with or without a blue light filter. A significant (*p* < 0.05) decrease in angiogenin secretion was observed under white-light exposure when compared to dark conditions and a significant (*p* < 0.05) increase in bFGF secretion was observed under white light exposure when compared to dark conditions. However, although VEGF secretion was increased under white light exposure, this increase was not found to be statistically significant. Analyses were conducted using Welch’s unpaired t test. When assessing the effect of a blue light filter on the secretion of angiogenic factors following white light exposure, a significant (*p* < 0.05) increase was observed in angiogenin secretion levels. Although bFGF and VEGF levels were decreased in the presence of a blue light filter, this decrease was not statistically significant. (NS: non significant; * = *P* ≤ 0.05; ** ≤0.01)

On the first experiment, a significant (*p* < 0.05) decrease was observed in angiogenin secretion levels following white light exposure (compared to dark conditions). Increases in both bFGF and VEGF were observed when RPE cells were exposed to white light compared to dark conditions (normoxia); however, only the increase in bFGF was significant (*p* < 0.05). The effect of blue light filtering was also assessed. A significant (*p* < 0.05) increase in angiogenin levels was observed within supernatant samples collected from RPE cells exposed to white light with a BLF, compared to samples collected from RPE cells exposed to white light without a BLF. Light-induced secretion of bFGF was decreased in RPE cells exposed to white light in the presence of a BLF compared to cells exposed to white light alone; however, this finding was not statistically significant. Although a slight decrease in VEGF levels was observed with blue light filtering compared to white light exposure, this decrease was also not statistically significant (Fig. 1).

**Fig. 2:**
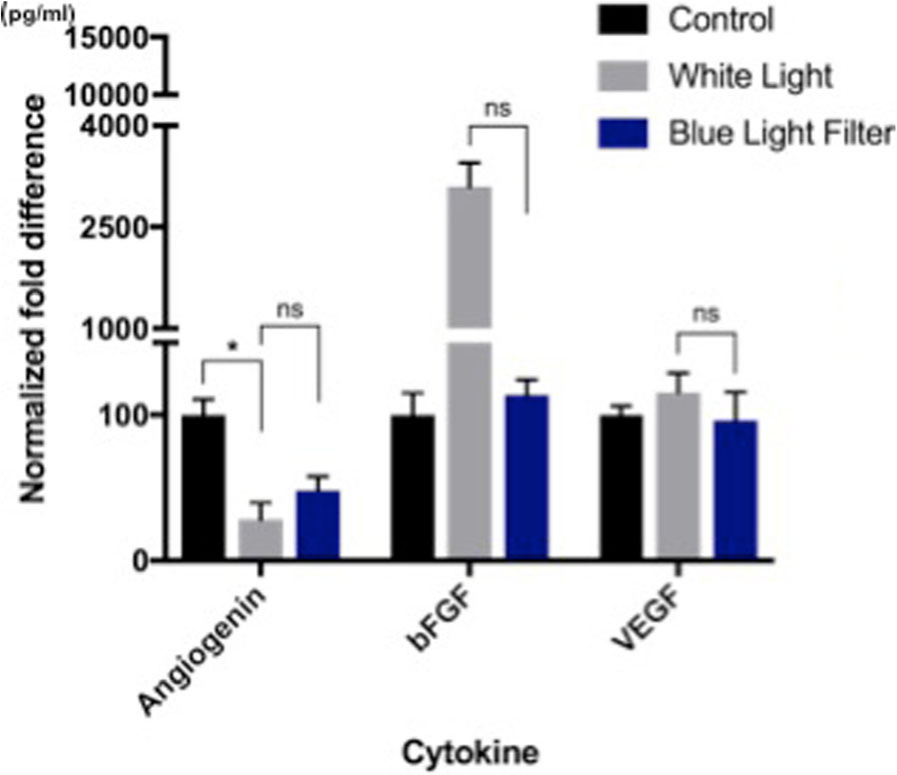
Experiment 2: Light exposure under hypoxia - Secretion levels of angiogenin, bFGF, and VEGF from supernatant samples of RPE cells grown under hypoxic conditions in the dark, or under white light with or without a blue light filter. Trends in the secretion of angiogenin, bFGF, and VEGF under hypoxic conditions were similar to those observed under normoxic conditions; however, none of the trends were found to be significant. (NS: non significant; * = *P* ≤ 0.05; ** ≤0.01)

On the second experiment, it was determined whether the effects of BLFs on the secretion of angiogenin, bFGF, and VEGF were maintained under hypoxic conditions. As seen under normoxic conditions, a significant (*p < 0.05)* decrease in angiogenin levels was observed upon white light exposure compared to dark conditions. A slight increase in angiogenin levels was seen when RPE cells were exposed to white light with a BLF; however, this was not found to be significant. In addition, a decrease in light-induced bFGF and VEGF secretion was observed with blue light filtering; however, these findings were also statistically insignificant (Fig. 2).

**Fig. 3:**
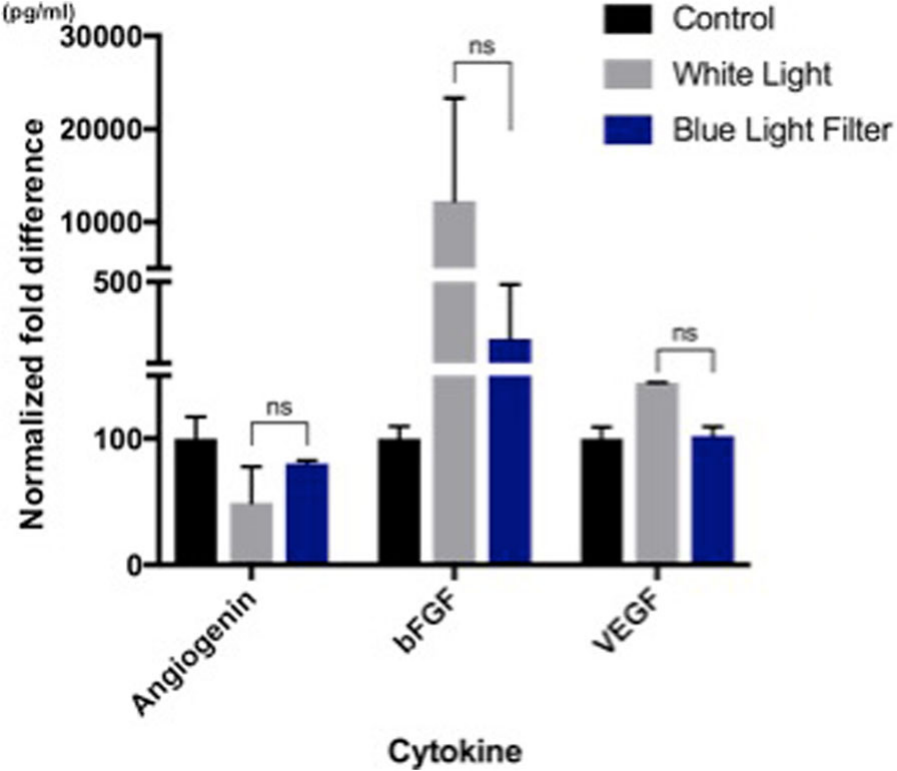
Experiment 3: Light exposure under normoxia when pretreament with lutein - Secretion levels of angiogenin, bFGF, and VEGF from supernatant with or without a blue light filter. Trends in the secretion of angiogenin, bFGF, and VEGF following lutein pretreatment were similar to those observed when RPE cells were grown under hypoxic and normoxic conditions; however, none of the trends were found to be significant. A slight increase in angiogenin levels was seen when RPE cells were exposed to white light with a BLF; however, this result was not found to be significant. In addition, decreases in light-induced bFGF and VEGF secretion were observed with blue light filtering; however, these decreases were also insignificant. (NS: non significant; * = *P* ≤ 0.05; ** ≤0.01)

On the third experiment, the effect of blue light filtering was also assessed when the cells under normoxic conditions were pre-treated with lutein. Trends in the secretion of angiogenin, bFGF, and VEGF following lutein pretreatment were similar to those observed when RPE cells were grown under hypoxic and normoxic conditions; however, none of the trends were found to be significant (Fig. 3).

## Discussion

In the present study, we investigated the effects of blue light filtering on the secretion profile of proangiogenic cytokines by RPE cells. To the best of our knowledge, this is the first study to demonstrate that following light exposure, angiogenin secretion by RPE cells is decreased, and this decrease is abrogated with a blue light filter. We also demonstrated that, although not significant, this trend is maintained when RPE cells are grown under hypoxic conditions and when pre-treated with lutein.

Although the etiology of AMD is not well understood, there is strong evidence indicating that retinal hypoxia plays a significant role in retinal neovascularization [21, 22]. On the other hand, lutein-containing supplements have been found to be beneficial and protective in slowing the progression of AMD [23–26]. We therefore sought to determine whether the effects of blue light filtering are maintained when RPE cells are exposed to hypoxic conditions or when pre-treated with lutein. We focused our investigation on angiogenin, VEGF, and bFGF given that their secretion was found to be above the limit of detection as indicated by our array system.

The role of angiogenin in the aging eye and in choroidal neovascularization is poorly understood. One study evaluating the histopathologic secretion of angiogenin in post-mortem control and AMD donor eyes noted similar secretion patterns, however no conclusive statement can be reached given the small sample size of three AMD eyes used in the analyses [27]. Results from studies assessing angiogenin levels in other ocular malignancies, namely diabetic retinopathy (DR), have been contradicting. Marek et al. (2011) described an inverse relationship in the concentrations of VEGF and angiogenin in patients with DR [28]. The authors observed a decrease in angiogenin and an increase in VEGF concentrations in both serum and vitreous samples from patients with DR compared to healthy individuals [28]. This is in accordance with our results demonstrating an inverse relationship between VEGF and angiogenin secretion levels following light exposure. Our group has also previously demonstrated an upregulation in angiogenin secretion with an expected abrogation in VEGF secretion following anti-VEGF treatment of RPE cells [20]. Our combined results support the hypothesis of a compensatory mechanism that is likely activated in response to decreased VEGF levels. To the best of our knowledge, this is the first report describing the differential secretion of angiogenin by RPE cells in response to a blue-light filter in vitro. Additional studies evaluating the secretion pattern of angiogenin in normal and AMD eyes, and the mechanisms underlying its down-regulation by RPE cells following light exposure are required.

Our results also demonstrated that blue light filters prevent light-induced up-regulation of bFGF and VEGF secretion to near normal levels. Although not significant, this trend is also observed when RPE cells are grown under hypoxic conditions and when pre-treated with lutein prior to exposure to experimental conditions. Unlike angiogenin, the proangiogenic effects of VEGF and bFGF in ocular neovascularization are well documented in the literature [12, 29–33]. It is also well established that VEGF and bFGF levels are increased in hypoxic conditions as a means of promoting neovascularization of ischemic tissues [34–36]. Our data on VEGF secretion are in accordance with previous reports demonstrating VEGF up-regulation by RPE cells following light exposure and its inhibition when cells are cultured with a blue light-filtering intraocular lens [37].

A recent study by Kishimoto et al. (2005) was first to describe the interplay between VEGF, bFGF, and angiogenin [38]. The authors demonstrated the important role that angiogenin plays in mediating VEGF- and bFGF-induced angiogenesis. Specifically, the authors showed that angiogenin is indeed required for VEGF and bFGF to stimulate angiogenesis [38]. In our study, we observed no changes compared to control in light-induced secretion of VEGF and bFGF with a blue light filter; however, there was a concomitant increase in angiogenin production. Thus, we hypothesize that RPE cells increase angiogenin secretion as a means to compensate for the protective effects of blue light filters. As anti-VEGF therapy is the gold standard treatment for neovascular AMD, it may be of value to further investigate the effect of anti-VEGF treatment on angiogenin secretion and action in the eye.

The detrimental effects of blue light on the viability of RPE cells are well documented in vitro and in vivo [39–44]. Blue light filters have been shown to protect RPE cells from light-induced damage and apoptosis in RPE cell cultures [10, 11, 45]. To the best of our knowledge, this the first study investigating the secretion of pro-angiogenic cytokines by blue-light exposed RPE cells under normoxia, hypoxia, and lutein-pretreated conditions. Our findings complement the growing body of evidence on the protective effects of blue light filtering IOLs, and suggest that these benefits may be maintained even when patients have dry AMD and are consuming lutein-containing supplements. It should be noted that the current study is not without limitations, and the previous statements require confirmation through additional in vitro and in vivo studies. For one, the light source used in our study yielded a range of light intensity albeit narrow, and may not mimic life-long exposure to blue light. Experts differ as to the exact wavelength of blue light; therefore it is difficult to be accurate when intending to block this specific wavelength range. Despite utilizing a yellow filter almost selective in blocking blue light, we must assume that minimal percentage of green light was also blocked, which we recognize as a limiting factor of our study. Another limitation of our study is the total number of replicates that may have prevented the attainment of statistically significant data points.

## Conclusions

Our findings support the position that blue light filtering affects the secretion of angiogenic factors by RPE cells under normoxic, hypoxic, and lutein-pretreated conditions in a similar manner. These results support growing evidence on the protective effects of blue light filtering IOLs and suggest that blocking effect on the blue wavelength light may be a benefit in patients with non-exudative AMD, also when consuming lutein-containing supplements. The cellular responses of RPE cells following blue light exposure warrants further investigation.

## Abbreviations

AMD: Age-related macular degeneration
ANG-2: Angiopoietin
AREDS2: Age-related eye disease study 2
bFGF: Basic fibroblast growth factor
BLF: Blue light filter
DMEM: Dulbecco’s modified eagle’s medium
DR: Diabetic retinopathy
EGF: Epidermal growth factor
FBS: Fetal bovine serum
HB-EGF: Heparin-binding EGF-like growth factor
HGF: Hepatocyte growth factor
IOLs: Intraocular lenses
PDGF-BB: Platelet-derived growth factor
PIGF: Placental growth factor
RPE: Retinal pigmented epithelial cells
SEM: Standard error of the mean
UV: Ultraviolet
VEGF: Vascular endothelial growth factor
